# Healthcare Service Providers’ Perspectives on Sociocultural Aspects Affecting Weight Management Activities Amongst People with Obesity in Taiwan—A Qualitative Study

**DOI:** 10.3390/nu16101540

**Published:** 2024-05-20

**Authors:** Jodie Leu, Kuo-Chin Huang, Pey-Rong Chen, Wen-Harn Pan

**Affiliations:** 1 Population Health Sciences, National Health Research Institutes, Miaoli 350, Taiwan; 2 Department of Family Medicine, National Taiwan University Hospital, Taipei 100, Taiwan; bretthuang@ntu.edu.tw; 3 College of Medicine, National Taiwan University, Taipei 100, Taiwan; 4 Department of Dietetics, National Taiwan University Hospital, Taipei 100, Taiwan; prchen@ntuh.gov.tw; 5 Institute of Biomedical Sciences, Academia Sinica, Taipei 115, Taiwan; pan@ibms.sinica.edu.tw; 6 Department of Biochemical Science and Technology, National Taiwan University, Taipei 100, Taiwan

**Keywords:** barriers, food, healthcare service providers, mental health, obesity, physical activity, sleep, weight loss and maintenance

## Abstract

The prevalence of obesity and morbid obesity in Taiwan has risen sharply in recent decades, as in other parts of the world, necessitating urgent action to prevent and curb its detrimental effects. Asian populations are susceptible to the repercussions of obesity at a lower body weight. A higher BMI is associated with more frequent outpatient visits, in-hospital admissions, higher medical costs, and a lower quality of life. However, effective weight management approaches are unlikely to be maintained in the long term without assimilation into daily lifestyle practices. This qualitative study, based on semi-structured interviews with 14 doctors, dieticians, and nurses who work to control the weight of people with obesity, explored and identified multilevel barriers in the context of daily life to improve the efficacy and execution of weight management strategies. They considered diets, physical activity, and sleep as key weight management activities. The cultural and psychosocial aspects of daily life were observed to have an impact upon weight management, particularly family conflicts due to cultural dynamics and socially and culturally reinforced food practices. To improve population weight, less-recognised aspects need to be addressed alongside the inclusion of mental health specialists in weight management protocols and policy interventions to minimise obesogenic practices and create environments conducive to weight management.

## 1. Introduction

As in other parts of the world, the prevalence of obesity and morbid obesity in the Taiwanese population rose sharply between 1993 and 2014, from 11.8% to 22.0% and 0.4% to 1.4%, respectively [1]. While Asian populations are not perceived as having an obesity epidemic, increasingly sedentary ways of living and the adoption of unhealthy dietary practices has led to rising obesity rates. Moreover, the Asian population is susceptible to the repercussions of obesity at lower body weights than other populations [2]. A high body mass index (BMI) is one of the leading five risk factors for disability-adjusted life years in Taiwan [3]. Consequently, BMI categories for Asian populations are adjusted to lower figures [4] with a BMI of 27 and over being considered obese in Taiwan [5] in comparison to the World Health Organisation’s cut off of 30.

Obesity has complex and multifactorial causes [6]; thus, it is important to carefully examine every aspect of healthcare service delivery to ensure the efficacy of weight management approaches. Taiwan has a single payer system of national health coverage that has allowed for affordable and accessible medical care and medication since its inception in 1995 [7]. With 92.6% of all hospitals, clinics, and healthcare facilities in Taiwan contracted as providers, the National Health Insurance (NHI) system allows patients to access doctors, including specialists, easily with relatively short waiting times [7]. However, an increasingly higher BMI is associated with more frequent outpatient visits, in-hospital admissions, higher medical costs [8], and a lower quality of life in comparison to non and less obese populations [9]. Additionally, because the healthcare system is set up for doctors to earn income through patient consultations, drug prescriptions, and minor procedures, there is a tendency for short consultations times, which may have an impact on doctor-patient relationships and their ability to deal with complex problems and also increase doctor shopping and medical costs [10]. 

At present, interventions utilising non-surgical and non-pharmacological means have limited long-term success. A systematic review revealed that the majority of individuals with obesity did not successfully maintain their weight loss for at least 3 years post-intervention with trends showing a gradual return towards pre-intervention weight [11]. There has also been increasing evidence that non-surgical options can be effective, especially when interventions are multidisciplinary, incorporating nutritional, physical activity, behavioural, psychological, and lifestyle interventions for weight loss [12,13,14]. However, many interventions fail to bridge the gap to assimilate learned habits and behaviours into patients’ lives outside of the interventions, particularly as lifestyle factors such as foods and sociability are strongly tied to and influence food practices [15]. For example, unchanged dietary practices and social factors post-bariatric surgery may result in weight regain [16,17]. Thus, findings from existing literature demonstrate the need to integrate behavioural changes and lifestyle factors as they are key to promoting and maintaining weight loss. While aspects such as a lack of time and the cost of foods have been recognised as common barriers to weight management, there is also a need to understand cultural aspects related to weight loss as each population may experience weight management differently. For instance, among the American Latinx community, there is a need for culturally appropriate nutrition education as adaptation to mainstream diets, i.e., modern diets associated with convenience, has led to the loss of traditionally healthy habits [18]. Literature from Australia and Europe also highlights increasing support for holistic care aligned with the needs and wants of people with morbid obesity [19,20]. 

Thus, to improve weight management care in an Asian context, it would be valuable to gain insight into the views of the healthcare service providers (HSPs) who help people with obesity (PwO) to manage their weight to explore and identify factors that impact weight management. To date, there is limited literature on the experiences of HSPs on weight management in Asia. There is only one existing study conducted in Malaysia on the experiences of community pharmacists and how they can play an important role in weight management; however, barriers exist in terms of providing private consultation rooms, a lack of time due to workloads, remuneration for longer consults, and training on weight management strategies [21]. To our knowledge, this is the first study exploring the perceptions and experiences of HSPs in an East Asian context to gain insight into current intervention methods, the weight loss journey of PwO and the extent to which cultural and psychosocial factors play a role in weight management activities. This study is part of a larger qualitative study that also seeks to explore the lived experience of PwO and their experiences with weight loss. The findings of the study can help inform future weight loss efforts in Taiwan and East Asian populations by highlighting multilevel lifestyle barriers in weight management activities to address and provide insight to better HSP-PwO interactions to improve weight management outcomes and care for PwO.

## 2. Study Design

A qualitative approach was taken to explore and gain insights into the perspectives of HSPs and their experiences with PwO [22]. Ethical approval was attained from the relevant ethics boards at National Health Research Institutes, Taiwan (EC1090804-E) and Academia Sinica (AS-IRB-HS 02-20027). The Consolidated Criteria for Reporting Qualitative Studies (COREQ) checklist was followed for the preparation of this paper [23].

### 2.1. Participant Recruitment

Recruitment took place between 20 November 2020 and 12 May 2021. E-mails with the study information were sent to members of three relevant professional associations: Taiwan Dietician Association, Taiwan Medical Association for the Study of Obesity, and the Taiwan Society for Sports Nutrition. Purposive sampling was used to recruit participants who were knowledgeable and experienced in the study matter [22]. Snowball sampling was also used to recruit eligible participants through existing contacts and participants. Twenty healthcare service providers in active practice, between the ages of 20 and 64, expressed interest in joining the study. However, six were ineligible as they were not located in Taipei or did not directly work with PwO. A total of two doctors, three nurses, and nine dieticians who worked in hospitals and private clinics and businesses with at least 2.5 years of experience providing services to PwO were recruited. No participants withdrew from the study. 

### 2.2. Data Collection

Face-to-face semi-structured interviews lasting approximately one hour were conducted in an enclosed meeting room or consultation room at participants’ workplaces or at a café in Taipei. Prior to the interview, the nature and purpose of the study was described along with participants’ right to withdraw from the study. To ensure consistency, all interviews were conducted in Chinese by the first author, a female postdoctoral researcher with more than 8 years of experience in qualitative research at the time of the study. JL had no prior relationship with the participants. Following each interview, participants were offered NTD500 (~USD17) convenience store vouchers to thank them for their time. Fieldnotes were also recorded by the interviewer following each interview. All interviews were audio-recorded except for one where the recorder malfunctioned, resulting in extensive fieldnotes being taken right after the interview ended to recall all of the interview content. Data saturation was reached when no novel information emerged [24]. No member checking occurred to minimise participant burden. The interview guide was pilot tested with acquaintances who fit the recruitment criteria before the first interview took place.

### 2.3. Data Analysis

Interviews were transcribed in Chinese by a professional transcription company. The first author checked and translated the bilingual transcripts. In the context of the study, and due to a direct translational approach, it should be noted that the use of the word ‘fat’ was often used as a descriptor instead of in a derogatory manner unless HSPs were quoting, or paraphrasing, stigmatizing words witnessed or shared by the PwO they interacted with. PWH checked three bilingual transcripts for quality control. The process of translating the transcripts allowed the first author to engage with the data and make notes to aid with analysis. Inductive and deductive thematic analyses were conducted to gain insight into themes identified from the literature while allowing for novel themes to appear [25,26]. The first author coded the transcripts using Atlas.ti 9 Windows. The broad themes were discussed with the co-authors who were experienced health researchers (WHP, KCH and PRC), two of whom were HSPs practicing in family medicine (KCH) and dietetics (PRC) and had experience working with PwO. The socioecological model (SEM) provided a framework for the analysis as the coding revealed various themes resonating with the model’s multilevel factors at the individual, relationship, community, and societal levels. The SEM was derived from Bronfenbrenner’s ecological model of human development [27] and has undergone several adaptations for various applications such as in public health [28] and violence prevention [29]. The SEM posits that health is influenced by proximal and distal interactions between the individual, community, and their environment—which need to be considered in attempts to promote health-promoting practices. 

## 3. Results

### 3.1. Participant Characteristics

The study included 14 HSPs who had experience helping PwO with weight loss (Table 1). Their experience with PwO spanned from at least 2.5 years to 25 years. Globally, dietetics and nursing are commonly perceived as pink-collar jobs which was reflected amongst the participants who were predominantly women [30,31]. At the time of interviews, all HSPs interviewed were working at tertiary hospitals or in private clinics and businesses such as those providing services for weight loss and well-being and fitness centres.

**Figure 1:**
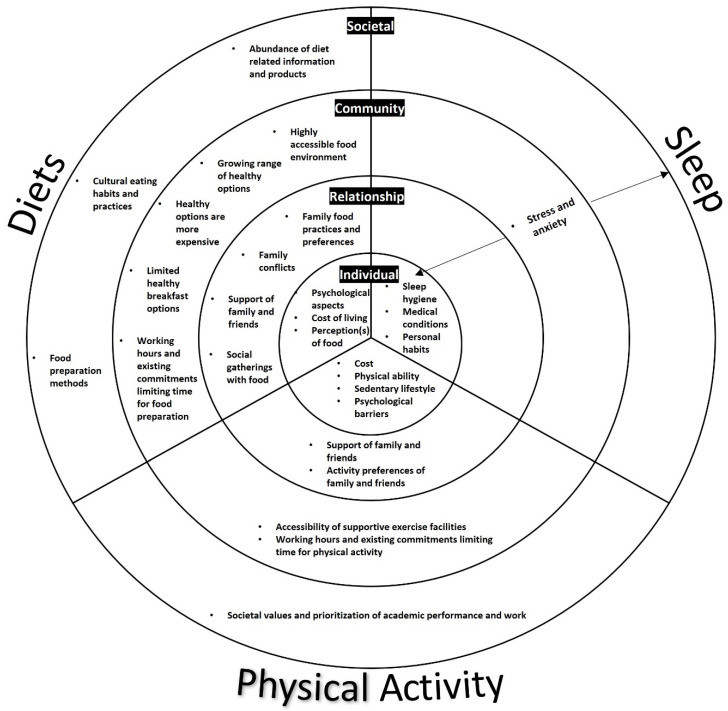
A conceptual model illustrating multilevel aspects perceived by healthcare service providers regarding diets, physical activity, and sleep that impact weight management among people with obesity.

The HSPs worked in a range of roles from treating PwO with and without pharmaceutical or surgical means, providing health education to manage diabetes, dietary consultations for non-surgical and surgical weight loss, and personal fitness. Three of the key weight management activities targeted by HSPs are diet, physical activity, and sleep. The SEM is used as a framework to present multilevel factors which the HSPs considered to impact each of the weight management activities and should be tackled in weight loss efforts among PwO (Figure 1). The quotes presented in this paper are labelled with a quote reference number corresponding to the original Chinese text provided in Supplementary Materials Table S1.

### 3.2. Theme 1: Multilevel Factors Influencing Diets

#### 3.2.1. Individual Level

The HSPs perceived several individual-level aspects which influenced the dietary habits of PwO. First, an individual’s perception of dieting and their psychological state influences whether they were receptive and able to adhere to dietary changes. Many HSPs observed that PwO perceived dietary changes and dieting as restrictive and unenjoyable, as summarised by HSP12 (dietician, private industry, 3 years of experience) “*…they may think that if [they] want to lose fat, it must be hard work, certainly can’t eat things. They will set themselves up with a difficult threshold. Like,* “*I just can’t ever eat cake again*”*, and so on*”. (Q1). In addition, HSPs also frequently identified and addressed PwO partaking in emotional eating that often resulted in an excessive intake of food. Commonly identified drivers were stress and anxiety derived from work, studies, and interpersonal relationships and, at times, from being in denial of their weight and health conditions. Consequently, HSPs may try to suggest eating less unhealthy food items or finding healthier alternatives. HSPs may also spend more time during consultations addressing emotional eating and suggesting alternative stress-relieving outlets that are not food related.

*…a lot of time is spent on solving emotional problems…whenever they face pressure, they can eat 2–3 bowls of white rice and one entire rectangular bar of cake…you know they are definitely, um, psychologically related problems, definitely not, um, physiological needs…But this process is very long because [they] keep on continuing the cycle, then you have to continuously talk to them, how to adjust their attitude, how to think, they may sometimes think of giving up, we have to try hard to encourage them.*

(Q2)

- HSP5: dietician, female, private clinic/business.

Income, working hours, existing commitments, and whether the PwO had a habit of cooking, and living conditions were also perceived to impact upon the types of foods PwO purchased and consumed. For instance, PwO may live in an ensuite room with no access to cooking facilities. Often, a mix of the aforementioned factors resulted in a reliance on commercial food items. The HSPs would take these lifestyle factors into consideration and attempt to suggest convenient and affordable healthy options. However, healthier food items are generally more expensive unless one knows where and how to access and assemble affordable healthy options and alternatives.

*…. For example, if they have eaten [a healthy lunchbox] before. I will ask them, for example, you, what do you think this kind of lunchbox concept is selling? It’s expensive but it isn’t just the price of the ingredients. It’s not expensive because of the ingredients but the concept…Well, today I do not have a lot of money, and I cannot buy this kind of lunch, then I went to 7–11, how do I achieve lowGI? It’s still possible!*

(Q3)

- HSP9: dietician, based in a hospital, 10 years of experience, describing how they would guide PwO on how they can access and assemble affordable meal items mimicking healthy concepts present in the healthy lunchboxes.

#### 3.2.2. Relationship Level

At the relationship level, familial food practices and preferences also influenced the dietary habits of PwO and their adherence to recommended dietary changes for weight management, such as the amount and quality of food and how food is prepared and eaten at home. As summarised by HSP8 (nurse, hospital, 20 years of experience), “*…their family’s habits are similar, for many people it’s not only themselves that’s fat, it’s the whole family that’s pretty fat* [chuckles]” (Q4). HSPs described common practices stemming from people’s preference for fresh ingredients and freshly cooked foods that lead to surplus food intake due to finishing dishes at each meal to avoid packing leftovers. The HSPs felt that these practices may be attributed to a sense of minimising food wastage, due to socio-economic backgrounds or concepts passed on from older generations that have experienced food shortages and economic hardship, such as during wars. Additionally, some households’ habits may have limited or non-existent cooking due to several reasons, such as a being time-poor and work schedules, contributing to norms that normalises a reliance on commercial food items throughout the life course. 

The support of people with whom PwO share a residence is also important. For instance, mothers attempting weight loss by cooking healthier dishes may face conflicts if family members dislike the dishes, resulting in lower motivation and inability to make consistent dietary changes.

*…maybe when she [mothers] goes back home, they will cook all the food really healthily, and then, and then, also have some husbands say “you eat what you cook, is this food edible? It’s so unpalatable, like dog poop!” Like this, then because of the family’s opposition, maybe in terms of her motivation, maybe will be influenced…*

(Q5)

- HSP8: nurse, hospital, 20 years of experience.

In other families, PwO may have to negotiate healthier cooking methods and dishes with family members that usually prepare the food at home. For some, these relationships may be fraught with tensions such as between mothers-in-law and daughters-in-law. Considering these social situations, dieticians in particular try to suggest ways for PwO to maximise their chances to have a healthier diet, at times sharing their own personal experiences. For instance, HSP9 (dietician, based in hospital, 10 years of experience) would share her experiences with PwO, such as telling mothers-in-law that the excessive use of cooking oil affects their weight loss or coming up with an excuse. HSP9 shared that when she lived with her in-laws, she would rinse leftover dishes with hot water to remove the oil and tell her mother-in-law that she was doing so to save time and electricity. 

HSPs perceived supportive family members to be important as they can facilitate conducive weight loss environments by preparing healthy foods and providing gentle reminders. Conversely, harshly critical family members were perceived to be unhelpful. When possible, HSPs offer PwO suggestions on how they can ask their family members to rephrase their reminders to be more supportive.

*Police-like family members [who closely monitor you], when you are eating, “You’re eating again, aren’t you losing weight? How can you eat this thing?”, this is called policing. Maybe if your family is nicer, tell them or ask them to say, “Oh, you eat this now…Then let’s take a look”…or maybe say “Are you really hungry?”, “Do you need me to help you see if you want to [know] how to control or something”, that is different, the words and communication words are different, so it’s different on the mentality of the patient.*

(Q6)

- HSP7: dietician, based in a hospital, 25 years of experience.

Similarly, unsupportive situations may also occur at social occasions where acquaintances and friends dissuade PwO from eating healthily. Social pressure can also result in PwO feeling uncomfortable about changing their food practices in others’ presence. For instance, being hesitant to choose healthier food items that were perceived as ‘uncharacteristic’ of them and wanting to experience the same foods with their friends and family during social and celebratory occasions. Entertaining and socialising for work purposes also often occur over communal meals where PwO may feel pressure to conform to the food preferences of their business partners. Festive seasons, particularly during the winter months, were often described as periods when many gain weight due to the higher frequency of food-based social gatherings. HSP10 (dietician, private clinic/business, 2.5 years of experience) listed the type of events occurring during winter in addition to people’s own social gatherings, “*from December to one month after the Lunar New Year* [usually occuring between mid January to mid February], *because after all, at this time it’s Christmas, New Year’s Eve, year end banquet* [traditional annual company event to show appreciation for staff]*, New Year, spring feast* [common company event after Lunar New Year], *wow! All celebrations*” (Q7). While HSPs often encourage PwO to share their intention to lose weight with friends and family to gain support, they are aware that there may be people and situations that hinder weight loss attempts. The responses of HSPs suggest that alternative channels of support may be needed such as through peer groups in weight-loss interventions. 

#### 3.2.3. Community Level

Due to the factors previously mentioned, the HSPs observed that a high proportion of PwO consumed commercially prepared foods. Additionally, the proliferation of food delivery services in the past few years has increased the accessibility and convenience of commercial foods. As surmised by HSP7 (dietician, hospital 25 years of experience), “*you see all the streets are filled with [food delivery workers]*” (Q8). However, the HSPs expressed mixed perspectives on whether the food environment, which was perceived as being highly accessible, affordable, and abundant in food options, was conducive to weight loss. 

While some HSPs felt that an abundance of choice meant that it was relatively easy to eat healthier, most mentioned the proliferation of easily accessible but unhealthy food choices and establishments, such as bubble tea shops. HSPs perceptions regarding the accessibility of healthy options also differed between meal types. Almost all HSPs felt that although breakfast items were easily accessed, options were limited and considered unhealthy and excessively oily, resulting in difficulties in suggesting healthier alternatives. Examples of commonly mentioned breakfast items were pre-made sandwiches made with processed meat and condiments, egg crepes, fried fritters, and flatbreads that were often paired with sweetened beverages such as tea with creamer or sweetened soy milk. Meanwhile, for lunch and dinner, there were more healthier options and alternatives to suggest. However, food options may be limited to the types of foods available within the vicinity of people’s abode and workplaces. 

HSPs also perceived growing health consciousness amongst the general population driving the sale and range of healthy items that are available from various commercial food outlets in recent years which has helped improved accessibility of healthier food options. However, healthier food items tend to be more expensive in comparison to other food items. A common example was the increase in takeaway shops selling lunchboxes with healthier items and healthy meal concepts which were often priced several times higher than the average lunchbox. In comparison, the average lunchbox which is considered an affordable and filling meal option is heavy on refined carbohydrates, salt, and sugar, and often includes fried items. Thus, a commonly recommended affordable and customisable option were ubiquitous neighbourhood self-serve buffet style shops. These shops generally provided a variety of dishes, including different types of vegetables cooked in various ways, thus providing more vegetable options to accompany staple food items. Some shops may prepare food with more oil and seasoning than others. Healthy choice items at convenience stores were also another option, particularly for time-poor individuals. Overall, customisable food options and items, such as sandwich fillings, are perceived as helpful for making healthier food choices. However, the suggestions and advice offered by HSPs to PwO often compete with personal food preferences, existing sociocultural practices, the higher price of healthier foods, and tempting food items in the food environment, preventing healthier choices and hindering weight loss.

*…We used to often say that when my patient wants to lose weight, they need a safe environment, but there are a lot of delicacies in Taiwan. Actually, this is very challenging for patients. Especially as Taiwanese cuisine has a lot of street foods* [Note: street foods tend to be classified as junk food due to preparation methods and are commonly referred to as a subsection of Taiwanese delicacies]. *Then, then this…to our patients, like older patients, in their impression of the food culture, their traditional diet, the traditional diet may be high in oil and sugar, but that is their past dietary experience, which they like, that will also make it difficult for patients to achieve their desired weight loss. But at the same time, these foods also have meaning to them.*

(Q9)

- HSP7: dietician, hospital, 25 years of experience.

#### 3.2.4. Societal Level

The HSPs pointed out some societal-level aspects, namely cultural norms and practices related to food, such as food preparation methods and eating practices, that were not conducive to weight loss and were perceived to be difficult to intervene. Although dietary principles may be similar cross-culturally, such as calorie control and nutrient density, the food types may differ and the application of healthy eating concepts from other countries and cultures may not translate well locally. For instance, vegetable oils are generally used for cooking (stir frying and deep frying) in Taiwan where there is a preference for hot meals. Meanwhile, in Western countries, vegetable oils may be used as part of a salad dressing. Moreover, differences in cultural eating practices and habits, particularly in communal settings, may mean that a different approach should be taken to promote heathier diets in Taiwan.

*Then also, the Westerners their diet is basically a plate, they’re like, this is one portion, that is one portion, they are using portions to calculate, this is different in Taiwan. In Taiwan we don’t have this plate habit, we just have a table of dishes, and everyone takes their own bowl and take what they want to eat…after I finish [the small serves of each dish], I grab more, finish eating, then grab more, so later I do not know how much I ate, it’s this kind of [eating] pattern…in the United States MyPlate can be promoted but can’t in Taiwan, but now we are starting to promote it, so what you want to eat, just take all [the dishes you want to eat] together [in one go] and then just stop, you cannot take anymore…a lot [of practices are] not the same…but the things in principle are the same, for example, to have low carbohydrate and high protein those, and so on…*

(Q10)

- HSP11: doctor, hospital, 20 years of experience.

Another societal level influence on weight management concerns the sources of easily available diet-related information. HSPs’ general perceptions towards these sources of information were that they were biased, incomplete, may be non-evidence based, and have an agenda behind them, such as promoting health supplements and weight loss pills. Thus, the HSPs find themselves correcting and educating PwO on dietary concepts, which are commonly acquired from the internet, to ensure that the weight loss methods are not detrimental to their health. Consequently, the HSPs understand that there is a need for them to stay abreast of the latest diet-related information and fads to make evidence-based judgements to provide timely dietary advice. The HSPs recognised that PwO may be attracted to these types of information, particularly those related to easy fixes. The HSPs attributed the fixation on easy fixes to PwO wanting to lose weight rapidly and easily and being unwilling or not ready to put in effort to lose weight by changing their diets and lifestyle. The effort of changing diets and lifestyle for weight management purposes is often referred to and thought of as ‘hard work’.

*…I often hear a person with a BMI greater than 35, often ask us one thing, they ask “Is there such a thing that you can lose weight without too much trouble, something with no need to change anything and then I can just lose weight?”, so that’s probably why there are so many weight loss methods on the internet like they would say that you don’t, don’t have to work so hard, and you can lose how many kilos…For people who want to lose weight, it is a need and desire, yes, but continuously, they actually have not been able to accept that, there is a need for hard work then they would be able to go through the process of losing weight…*

(Q11)

- HSP1: dietician, private clinic/business, 8 years of experience.

During consultations, PwO may share, discuss, or even argue with HSPs on the ‘proven’ effectiveness of weight loss methods they deem effective or had helped them lose weight in the past. Under these circumstances, the HSPs generally will not directly confront PwO to minimise conflict and maintain existing rapport. When PwO have a preferred method that they want to try, the HSPs would verify the method, sometimes with modifications to ensure the methods promote healthy weight loss. Here, HSP11 (doctor, based in hospital, 20 years of experience) summarises the approach the HSPs in the study had, “*so of course you must first clarify with them what they heard, some things are actually OK, then you just say OK, because the best weight loss method is the method that they can accept*”. (Q12) 

### 3.3. Theme 2: Multilevel Factors Influencing Physical Activity

#### 3.3.1. Individual Level

The HSPs commonly observed sedentary ways of living in conjunction with unhealthy dietary intakes as drivers of obesogenic lifestyles. As part of weight loss, the HSPs felt that it was important to start and increase physical activity and build a habit of exercise. However, they observed several individual factors influencing whether PwO take part in physical activity, such as physical ability. For individuals who suffered from severe obesity, HSPs might refrain from suggesting physical activity from the beginning and wait for diet-related weight loss to mitigate weight-related pain and injuries. Usually, a common suggestion was to start with low impact physical activity such as riding a bike or walking and increasing these opportunities, such as by parking vehicles further from their destination before progressing to a more strenuous exercise of their choice, such as running or circuit training. The HSPs understood that the cost of engaging personal trainers and joining gyms could reduce exercise options for PwO with limited budgets. Other barriers to physical activity that HSPs observed amongst PwO were a fear of unfamiliarity, internalised weight stigma, low self-confidence, and self-discrimination.

*Hmm…a person with heavier weight, just telling them to go into the gym, they, in fact, would be afraid, also [will be afraid of people seeing them], then “I don’t know how to use these things, looks very difficult. I don’t feel like this suits me”. I think that psychological fear…that would be scary, not to mention that towards their body, they still more or less will feel a little, “ah my weight is heavier [than others]”*

(Q13)

- HSP6: dietician, hospital, 9 years of experience.

#### 3.3.2. Relationship Level

HSPs also identified several relationship-level factors that deter PwO from taking part in physical activity. For many PwO, family commitments may be non-negotiable, such as taking care of children and the elderly, which can occupy time that could be utilised to partake in physical activity. While it may be helpful for PwO to join an exercise group or have family and friends to exercise with and incorporate exercise as part of their social gatherings, it may not always be possible due to the habits, preferences, and choice of social activities among PwO and their family and friends.

*…because long-term they would develop like, “my habit is now like this, so my family diet is like this, my friend’s diet is also like this, so long term-wise I don’t exercise, then you want me to suddenly have people who exercise in my life”, I think this may be relatively not so easy ah…I have encountered [a patient]…I told her to exercise. She said “Um…but I want to date my boyfriend, my boyfriend doesn’t want to exercise. The two of us want to go eat delicious food, then if I go to exercise, the two of us will have less time to date”*

(Q14)

- HSP6: dietician, hospital, 9 years of experience.

#### 3.3.3. Community Level

For many PwO, their working hours can make it difficult for them to incorporate physical activity on a regular basis. HSP6 (dietician, based in hospital, 9 years of experience) recalls her observations that “*because working hours may be from 8 to 6, and even in some industries, it will get past 7 o’clock, even later. So, for them, [after] going home to have a meal, in fact, it’s about time to have to rest*”. (Q15). Additionally, negative experiences, inclusive of weight stigma in public and communal spaces and personal preferences, can lead PwO to abstain from exercising in the presence of others, thereby limiting the types of physical activity options and locations. For instance, HSP10 (dietician, private clinic/business, 2.5 years of experience) shared that women may not enjoy going to gyms that often have a permeating smell of sweat and are male dominated—where many are grunting while exercising. With women-only gyms being a minority, women have less options for physical activity. In other instances, some exercise facilities may not be able to accommodate PwO, such as gyms where the equipment has weight thresholds. So, it was important for HSPs to suggest other forms of physical activity in suitable locations.

*[A patient] said, because many people told her to exercise. Many medical staff told her she must go, go to the gym to exercise, but she told me “it’s not that I don’t want to”…she felt very sad…because she said, [the gym] equipment is not for people more than one hundred kilograms, they’ll get injured…[I suggest she] do some exercises at home first. Then you go to the rehabilitation physician and ask them to teach you some steps. Then you can do it yourself. So, she later went to download some very simple body exercises from YouTube and they did it at home*

(Q16)

- HSP9: dietician, based in hospital, 10 years of experience.

#### 3.3.4. Societal Level

At the societal level, existing perceptions of physical activity can lead to a lack of long-term habits and disinclination towards exercise. HSPs frequently mentioned how the strong focus on academic performance in Taiwanese society has resulted in instances where exercise classes were not mandatory or removed from the curriculum in favour of being replaced by other classes and study sessions. For example, students may have their names marked for attendance and choose to self-study. In some places, “*…even elementary schools will sacrifice physical education to go study!*” (Q17) (HSP12: dietician, private clinic/business, 3 years of experience). Although the HSPs observed more exercise facilities opening in the past few years, which they attributed to people’s growing consciousness of health, they also shared existing generational perspectives towards exercise that can influence subsequent generations.

*I don’t know if it is the same as the early days of Taiwan, like my parents’ era…For them, there isn’t say any leisure to exercise. They feel like sports is recreational entertainment, which isn’t something that is necessary in life. In life, it’s just, go to work, come back, rest, and prepare for tomorrow, go to bed, go to work tomorrow*

(Q18)

- HSP6: dietician, based in hospital, 9 years of experience.

### 3.4. Theme 3: Multilevel Factors Contributing to Inadequate Quality Sleep 

The HSPs observed many PwO sleeping late and having an inadequate quality sleep which was perceived to interfere with factors related to weight loss, such as metabolism and food and activity practices. For instance, the HSPs noted that inadequate sleep led to a lack of physical activity and eating snacks to stay awake due to feeling tired. At the individual level, sleep may be influenced by medical conditions and sleep disorders, such as sleep apnoea, and medications. Personal habits can also contribute to bad sleep hygiene, such as using digital devices before bed and having a habit of sleeping late. The HSPs also described how sleep was influenced by stress and anxiety derived from individual, relationship, and community factors. For instance, stress from interpersonal relationships and work can lead to disrupted sleep.

*…most people sleep after midnight, or even 1 [am] eh!…then it will definitely affect your hormones, and on the other hand, people now have a lot of pressure in life, lots of work pressure, and also-, or bad long-term living habits, which leads to sleep quality getting worse and worse. And with our clinic’s experience, the most difficult to adjust is sleep…I do not know if it’s life stress or bad lifestyle habits, that is, that is, fiddling around or [thinking] “I think twelve o’clock is too early to sleep eh”…*

(Q19)

- HSP5: dietician, private clinic/business, 9 years of experience.

Amongst the HSPs, such as HSP5, most felt that sleep was difficult to adjust and intervene while some, like HSP3, felt that, among PwO without sleep disorders, “*most of them can try to adjust by themselves*” (Q20). Overall, it was difficult for HSPs to address sleep issues beyond referring PwO to relevant health departments, providing tips for sleep hygiene, and educating PwO on the benefits of sleep and its role in weight management. As shared by HSP10, the HSPs are unable to delve further as it was implied that stress and anxiety, which can also lead to emotional eating, may need to be dealt with by mental health professionals who may also be better equipped to help PwO deal with other issues such as weight stigma, self-discrimination, low self-confidence, and emotional eating triggers.

*Is it work? Home? Or is it your personal emotions? Or if it’s just due to breaking up and so on, then they will say it themselves. Then if you want to go a little deeper, to be honest, I really can’t help much?!*

(Q21)

- HSP10: dietician, private clinic/business, 2.5 years of experience.

However, all of the HSPs recognise that mental health stigma may deter patients from seeking mental health care. Additionally, the HSPs also commented on there being a shortage of mental health specialists, especially those who have experience or are specialised in obesity and the high cost of consultations, approximately 1000–1200 NTD (approximately USD31—38) per session which are not covered by the NHI, as barriers to access. 

## 4. Discussion

This study explored the perspectives and experiences of doctors, dieticians, and nurses that assist PwO with weight management. We found that HSPs perceived that a multi-pronged approach consisting of healthy diets, physical activity, and good quality sleepas important keystone activities that need to be maintained to help weight loss and long-term weight management. These findings echo the existing literature, supporting and demonstrating that multidisciplinary approaches are more successful for weight loss [12,13,14]. 

### 4.1. Interconnected Multilevel Barriers to Weight Management 

Using the socio-ecological model as a framework (Figure 1), the HSPs’ accounts revealed multilevel interconnected aspects, such as how individual psychological, psychosocial, and cultural aspects influence the keystone weight management activities which are also interconnected. There are interconnected aspects between diets, physical activity, and sleep at the distal and proximal levels. On an individual level, the economic background of PwO influences their ability to eat healthily and engage with more options for physical activity [32] which in turn can influence sleep. Economic factors have been continuously associated with obesity in other countries as well, contributing to the consistent emphasis on the provision of affordable healthy food environments and public amenities to promote more physical activity [33]. The psychological perceptions of PwO regarding the perceived difficulty of dieting and physical activity, internalised weight stigma, and their physical ability may be tackled by initially suggesting simple behavioural changes and options. On a relationship level, friends and family have a large influence on dietary and physical activity aspects. While supportive family and friends can be immensely helpful when PwO take part in weight management activities, our findings illuminate how the lack of support and the nature of social relationships impact advised dietary and physical activity changes. For instance, general formations of friendship groups are likely to be based on common interests as similarities breed connection [34]. By extension, within a social network, the chances of being obese also increase if an individual has a family or friend that is also obese [35], meaning PwO are likely to have friends and family who partake in similar lifestyle practices. Furthermore, on a community level, long working hours have been found to impact on an individual’s ability to take part in health-promoting behaviours [36]. However, these factors, in conjunction with non-interconnected influences, add another level of complexity to the barriers facing weight management activities, especially if they are long-standing issues that have continually resulted in PwO experiencing difficulties with their weight for extended periods of time. 

However, the HSPs understood that weight loss is not as simple as directing and instructing individuals on weight management activities. Instead, multilevel socioecological influences such as lifestyle, cultural, social, and psychological factors (Figure 1) have an impact on weight management activities, particularly as these influences vary between individuals and are likely to be longstanding and play a role in weight gain and difficulties with weight loss throughout the lives of PwO. Although HSPs exhibited flexibility in tailoring their weight management advice, with a focus on their area of expertise, they may not be adequately equipped to address multidisciplinary aspects, particularly mental health, and psychosocial and cultural practices. Our findings highlight the need to incorporate psychological expertise into weight management and the importance of finding culturally and socially sensitive ways to address and navigate socioecological influences to bolster comprehensive multidisciplinary efforts to assist PwO in better executing and integrating weight management activities in their daily lives. These findings support existing calls for a more holistic approach to be taken in addition to tackling existing known barriers to keystone activities, such as cost and time, given the complexity and multifactorial causes of obesity [6,32]. This is particularly important as weight loss is a complex experience among PwO and maintaining habits and behaviours learned from interventions in various real world settings is difficult [15] and weight regain due to the resumption of unhealthy lifestyle practices is common [11]. With the onus placed on PwO to execute weight loss activities once they leave consultations, there is also a continued need to address obesogenic factors in the community and environment to facilitate easier and successful weight management [33].

Currently, HSPs encountering PwO experiencing psychological, social, and cultural barriers to weight loss activities provide advice ranging from behavioural change suggestions to sharing, sometimes personal, anecdotes on how to overcome these challenges. While some aspects are perhaps more straightforward, such as suggesting accessible and affordable healthy alternatives to demonstrate how ‘easy’ dietary changes can be, other aspects such as cultural food practices in communal settings and promoting physical activity in the absence of supportive family and friends can be particularly difficult to target. 

### 4.2. Limitations, Barriers, Disinterest, and a Disinclination towards Physical Activity 

The HSPs agreed on common multilevel limitations and barriers that influenced the inclination motivation to participate in and expressions of interest in physical activity among PwO. These ranged from physical (in)ability, psychological perceptions, socioeconomic factors, social networks, community, and cultural and environmental factors. The combination of these influences led to varying degrees of disinclination and disinterest towards physical activity. For instance, physical ablity to exercise, perceptions and experience of physical activity, time, personal commitments, access to gyms, suitable exercise equipment at gyms, judgement free safe spaces to exercise, and supportive friends and family who were interested in physical activity. As noted by the HSPs, it was difficult to promote physical activity when PwO did not have friends and family who were inclined to exercise. Leu et al. [37] have found that, amongst Singaporean young adults, social networks and interests influence whether an individual takes part in physical activity; for instance, close acquaintances and joining groups with similar interests can help promote more exercise. Furthermore, weight stigma perpetuated by others in the community may increase the disinclination of PwO towards physical activity [38,39]. Additionally, the average annual hours worked in Taiwan came fourth at 2000 annual hours behind Singapore (2298), Mexico (2128), and Costa Rica (2073) in 2021 [40], thus highlighting issues regarding PwO having time for physical activity. In conjunction with existing personal commitments, such as caring for children, PwO may have even less time for physical activity. Furthermore, the prioritisation of educational and work performance throughout generations has meant that physical activity has not been cultivated as a regular and necessary activity to maintain health. In Singapore, another Asian country that also prioritises educational and work performance, tertiary education students prioritising their studies led to a lack of adequate physical activity [37]. Unless workarounds are found, these factors can have follow-on effects, such as PwO exercising only when certain conditions are met. 

Walking is often promoted as a simple, familiar physical activity that is feasible for most PwO and a workaround to psychosocial barriers. However, road safety in Taiwan is an increasingly critical issue at the community level due to existing infrastructure, motorist practices, and shared roadways. Every day, 8.3 people die from traffic accidents in Taiwan and the number of casualties and injuries from traffic accidents more than doubled from 229,666 in 2008 to a peak of 485,305 in 2020 [41]. In the same timeframe, pedestrian casualties and injuries increased from 11,149 in 2008 to peak at 16,946 in 2020 [42]. Overall, there is a need to improve road safety and the built environment to promote more physical activity in public spaces [33]. 

### 4.3. Multilevel Factors Influencing Diets

The multilevel and interrelated factors affecting diets are more complex than those affecting physical activity [6]. Barriers to a healthier diet that corresponded to those in the existing literature were also identified in this study, including personal economic circumstances [32], emotional eating [43], and psychosocial factors such as family food preferences and practices [44], and modelling food choices and intake based on their meal partners [45]. Thus, the HSPs found that, when providing dietary advice, understanding PwO and their needs was important for providing individualised approaches. However, psychological barriers and social and cultural practices are often nuanced and may be difficult to target. 

Psychological barriers, ranging from perceived difficulty and negative perceptions of diets to emotional eating among PwO, present varying levels of difficulty regarding how they are addressed due to the complexity and source of triggers. Emotional eating is described as a tendency to overeat in response to negative emotions, such as anxiety or irritability, and is more prevalent amongst individuals who are overweight or obese [43]. Difficulties in emotion regulation are associated with more weight regain due to emotional reasons ranging from loss of control to binge eating, lower perceptions of success in terms of maintenance, and few dietary strategies for maintaining weight [46]. Although HSPs may suggest non-food-related stress-relieving outlets, addressing emotional eating is an aspect that would require time and the incorporation of mental health specialists. Focusing on the dissemination of strategies related to healthier dietary intake without teaching skills to regulate emotions may not be effective amongst a group that experiences various emotions related to weight and weight loss [46].

Psychological aspects may also be further impacted by social and cultural factors which are difficult to intervene in as this may mean changing longstanding familial, social, and cultural practices and existing social relationship dynamics. As family food preferences and practices deeply influence daily meals [44], interactions between PwO and their families are important aspects to consider in weight management. While helpful family members may be sources of support for PwO, family members who may be well meaning but use inflammatory language appear to hinder weight loss attempts and progress. In Taiwan, families and relatives are top sources of stigma for PwO, alongside doctors [47]. Weight-based talks with family members and partners at different stages of life led to higher levels of distress [48] and unhealthy eating behaviours, particularly if the weight-related comments were hurtful [49]. Increasing links between weight stigma and unhealthy coping strategies, such as binge eating, have been found among PwO [50,51,52]. Similarly, Taiwanese young adults and adolescents experiencing weight stigma was associated with internalized stigma and anxiety, depression, eating disturbances, and unhealthy eating behaviour [47,53,54,55]. Additionally, there may be nuances that HSPs may not be privy to or be able to identify, such as stressors and family dynamics that present as barriers towards dietary changes. Even when HSPs are aware of culturally sensitive relationship-related barriers, such as conflicts over cooking methods in the home between mothers-in-law and daughters-in-law, it appears that HSPs may only share anecdotes about how others try to overcome these tribulations. In-law relationships have long been fraught with tensions, though living together brings the experience to another level of conflict, especially if relations are not on good terms [56]. As living with in-laws is a normative practice in Taiwan, this issue may be more prevalent. Thus, to address and alleviate these sociocultural barriers towards diets, mental health professionals specialising in family conflicts may help improve communication and find culturally acceptable ways to de-escalate heated situations to create a conducive weight loss environment.

When eating with others, people tend to model their food choices and intake based on their companions [45]. Additionally, eating with others is also intermixed with societal-level factors, such as cultural dining practices in a variety of social settings. In comparison to other Chinese-origin populations, such as Malaysian Chinese, the Taiwanese place more emphasis on the social function of food sharing occasions [57], which translates into more communal food occasions to facilitate the formation and maintenance of social bonds for family, friendship, and business [58]. Communal dining practices, such as the practice of shared dishes accompanying staple food items and finishing leftovers to minimise food waste, are also longstanding generational practices [58,59]. As with other Asian nations such as China, Japan, Korea, Vietnam, and countries with ethnic Chinese people as the majority, such as Singapore and Macao, in Taiwan, the ideologies of Confucianism are prevalent in every aspect of society [60]. The influence of Confucianism whereby hierarchical relations that are characterised by respect for elders, authority, and deference to social status are widely accepted norms. Under these social and cultural influences, PwO may struggle to go against existing social norms and dining preferences in their social group especially when eating with older family members and authoritative figures for work purposes. A systematic review of food choice among Chinese mainlanders and immigrants found that people would eat unwanted food or overeat to avoid conflict, maintain harmony within the social group, and show respect and gratefulness to the food provider [61], with some going as far as ignoring physical needs, even if they had type 2 diabetes [62]. Moreover, as part of East Asian business practices, communal meals with an abundance of food is characteristic of a host’s generosity and is important for the formation of a relationship [63] and the maintenance of relationships [64]. 

Furthermore, social capital can also influence eating practices through prevalent social norms, values, and attitudes in social networks. Zhang et al. [65] found that ethnic minority groups in China who transitioned from subsistence living to higher levels of living cared more about eating for fulfilment and pleasure and the intake of modern diets that are high in fats, salts, and sugars, contrasting existing studies where higher standards of living were generally associated with healthier diets. Consequently, they also found that bridging social capital, such as through social organizations, which cultivates the generation of ideas and innovations that would otherwise be unlikely among like-minded group members, such as family and friends, may be a method to address unhealthy eating practices. In relation to HSPs’ observations of homogeneous dining practices among PwO and their family and friends, social occasions may benefit from social interventions that promote healthier eating practices by changing detrimental psychosocial influences on communal dining practices. 

Within the existing highly accessible and affordable food environment, the convenience of purchasing cooked foods from convenience stores, food stalls, and food delivery services encourages the normative practice of eating food outside [66]. With high frequencies of eating out amongst the Taiwanese population [67], the advice offered by HSPs about where and how to purchase affordable healthier items reflects these practices. However, policy-level changes are needed to address the ubiquity of unhealthy items to minimise their intake and curb the effects of obesogenic food environments. While the Taiwanese government has targeted some aspects, such as regulating all-you-can-eat restaurants and limiting the advertisement and promotion of unhealthy foods, progress remains slow [68]. Furthermore, the policies do not address cooperative measures with food manufacturers and the foodservice industry to produce healthier items and dishes. Regulatory measures limiting the availability and accessibility of unhealthy food while addressing barriers towards access and affordability of healthy food items are important to support healthier for choices for PwO and the population [33]. Another aspect that HSPs mentioned was the need to stay informed about current trends in weight loss. As PwO are perceived as easily enticed by methods touting fast and effective weight loss, it is vital that these methods are screened to prevent detrimental outcomes. Evans at al. [69] found that, among PwO, many attempts at weight loss are often self-guided without using evidence-based strategies. In Taiwan, PwO, especially women, often sought anti-obesity drugs for treatment before going for professional treatment and often had unrealistic weight loss goals that resulted in high attrition rates from weight loss programs [70,71].

### 4.4. Multilevel Factors Influencing Sleep

HSPs’ perspectives on the importance and need to incorporate sleep in weight management is in line with growing literature supporting the incorporation of improving sleep for obese populations in addition to the focus on diet and physical activity [72,73]. Good quality sleep and adequate sleep are associated with greater weight and fat loss [74] and are important for body regulation related to BMI as inadequate sleep has been found to influence hunger hormones, resulting in feelings of unsatiety [75,76]. Sleeping at a regular time also helped with weight loss maintenance [77]. However, HSPs commonly observed that bad quality and inadequate sleep wreaked havoc on weight management. HSPs also had mixed perspectives on whether sleep could be intervened. Currently, PwO with sleep-related medical conditions interfering with their sleep are referred to related specialists. In the absence of medical conditions, HSPs generally try to persuade PwO about the merits of sleep, its role in weight management, and provide tips for sleep hygiene. 

The increasing use of technology, particularly interactive technological devices such as laptops and mobile devices, the internet, and social media has exacerbated detrimental sleep behaviours such as going to bed late and bad-quality sleep [78,79]. Moreover, there has been a growing prevalence of a worrying trend in sleep procrastination, where people go to bed later than intended without any reason [80]. Sleep procrastination has been shown to influence people’s sleep duration and is associated with low self-regulation [81]. The HSPs often mentioned PwO sleeping later due to using their mobile phones which results in a loss of sleep. These observations were consistent with a Dutch study which found that people went to sleep late due to deliberate procrastination where they felt they deserved time to themselves, mindless procrastination where they were preoccupied with evening activities, and strategic delay where they felt going to sleep later would let them fall asleep faster [82]. Similarly, Wang et al. [83] found that, among students at a university in Taiwan with a high use of social media, 61% categorized their sleep quality as poor and may be likely to develop social media addiction and sleep disorders. Additionally, noise pollution and its influence on quality of life and sleep has been increasingly recognized to have detrimental effects on human health, impacting upon general well-being, mental health, diabetes, and obesity [84]. However, where and what type of housing one lives in is constrained by other factors such as socioeconomic background [85]. Moreover, within the home, caring for children and invalid family members, and living conditions, particularly for women, may be other factors creating sleep disturbances that are difficult to alleviate [86,87]. 

In a general population sample of Taiwanese adults, the average nighttime sleep duration was 6.1 h with nearly half (46.6%) reporting poor sleep quality [88]. While it may suffice to incorporate sleep specialists in weight management, there are issues in implementation. Although sleep medicine in Taiwan is considered to be relatively new, being introduced nearly 30 years ago in the 1990s, it has developed rapidly through its expansion of specialist departments throughout medical institutions in Taiwan and publications. However, there is not enough equipment and staff to continue its development due to funding, resulting in issues such as physicians in sleep medicine focusing on their original medical expertise [89]. Furthermore, merely incorporating sleep medicine as part of the weight management process may not be adequate in addressing behaviours, the causes of stress and anxiety, and living environments that impact upon sleep. In the context of everyday living, apart from suggesting tips to promote better sleep hygiene, improving sleep quality remains difficult to target. Nonetheless, it is important to continue efforts to improve sleep as it has knock on effects on mental health [90], diets, and physical activity.

### 4.5. The Need to Address Psychosocial and Psychological Aspects Impacting on Weight Management

As everyone’s circumstances are different, an important aspect in weight management service delivery is gaining insight into the lived experiences of PwO, including lifestyles, practices, habits, and existing health conditions, to address barriers they face in weight management and to facilitate a person-centred approach. In current weight loss efforts, psychosocial aspects are rarely considered or targeted although they have been found to influence weight management activities. This study has identified common social and cultural barriers that HSPs observed impacting PwO in Taiwan. However, to be able to identify these barriers, there is a need for HSPs providing care for PwO to identify and resolve social and cultural barriers to weight management that may not be immediately obvious or nuanced. Though, these aspects may be easily overlooked due to limited consultation times [10] and doctors being a source of weight stigma [47]. Additionally, Weng et al. [91] observed that doctors in Taiwan may prioritise treating a patient’s disease and be results-focused, thereby ignoring patients’ subjective feelings, though years of experience and medical education helps improve emotional intelligence in patient care. However, with the prevalence of obesity on the rise, perhaps intensive educational units may be offered to HSPs to speedily improve their emotional intelligence. Additionally, there may be a need for HSPs to refer PwO to mental health specialists as it is increasingly apparent that psychological and psychosocial aspects appear to permeate many aspects of weight management and should be addressed among PwO. 

Currently, there are difficulties in accessing mental health services due to cost, waiting times, and the number of practicing mental health professionals in Taiwan [92]. Tu and Jin [93] have found that people who may require mental health services are reluctant to pay as visits to counselling psychologists are not covered by National Health Insurance (NHI) and private health insurance, which is supported by our findings, especially if PwO need to go regularly. Additionally, stigma toward mental disorders and lower levels of mental health literacy in Taiwan [94], similar to those in other Chinese communities [95], may prevent PwO from consulting with mental health professionals. These findings concur with a Canadian study with HSPs and PwO, reiterating the need to focus on mental well-being to help with weight loss efforts [96]. Existing research from Australia, England, and Europe demonstrate the need for holistic care [19,20]. The findings of a multinational study exploring obesity, public attitudes towards obesity prevention and psychological distress also support the idea that mental health issues need to be addressed when addressing overweight and obesity amongst populations [97].

### 4.6. Strengths and Limitations

This study adds to the limited existing literature that provides insight into the experiences and perspectives of HSPs who help PwO with weight management through a qualitative approach in an Asian context. The use of qualitative methods allows for the collection of deep descriptive data that illustrate the nuances in interactions between HSPs and PwO [25]. The study’s rigour was improved by pilot testing the interview guide prior to actual interviews. Data collected from all the HSPs were generally complementary and the study was deemed to have reached saturation when no additional data emerged [24]. Although, due to the nature of qualitative studies, the small sample size may not be generalizable to other populations. 

The small sample size may be due to few HSPs interacting with PwO with enough frequency to share their experiences as the prevalence of obesity is relatively low in Taiwan (22.0%) [1]. Furthermore, the small subset of recruited doctors and nurses could be due to difficulties in obtaining participation by doctors and healthcare service providers in research as they are time-scarce populations [98]. Additionally, this study was conducted during the COVID-19 pandemic in the months leading up to Taiwan’s first nationwide lockdown in May 2021 when HSPs, particularly doctors and nurses, were busy preparing for local outbreaks and helping on the frontlines. Selection bias during recruitment may have resulted in over presentation of HSPs who were more patient-friendly and were comfortable being asked about their thoughts and relationships with patients. Additionally, desirability bias and the Hawthorne effect could have led HSPs to present themselves in a more positive light. The shared aspects of service delivery were also not able to be verified without observing how consultations take place. However, the HSPs’ views concurred with a breadth of international research regarding weight loss among PwO and the experiences of other HSPs, such as the influence of sociability on food practices [15]. 

Nonetheless, the study provides insight into psychosocial and sociocultural factors impacting upon weight management and the experiences of HSPs in an Asian context. The findings of this study may also help inform the provision of weight management services in healthcare settings for East Asian populations in non-Asian countries. Furthermore, as social and cultural practices can have beneficial and detrimental impact upon weight management activities, future studies could incorporate these factors in addition to current attempts to measure social support for healthy lifestyles among PwO [99]. Such studies could work in conjunction with social prescribing to provide culturally appropriate social support for healthy lifestyles to prevent and curb obesity in various populations.

## 5. Conclusions

Our findings illuminate various aspects that present multi-level barriers towards three key weight management activities, diets, physical activity, and sleep, from the perspectives of HSPs that work with PwO. The findings reiterate and support the need for a more holistic approach that addresses cultural, psychosocial, and psychological aspects that impact weight management instead of the conventional focus on diet and physical activity. With the increasing prevalence of obesity in Taiwan, there is also a continued need for more training and emphasis on eliminating and alleviating weight bias, stigmatisation, and discrimination which could help reduce negative experiences faced by PwO in all aspects of their lives [52,100]. Additionally, there is also a need to incorporate mental health specialists to address long-standing psychosocial aspects that impact upon weight control and to include sleep as a weight management activity. The findings of the study can help inform weight management approaches among East Asian populations with obesity. There is a concurrent need for ongoing policy-level interventions to improve access and affordability of healthier food items and public amenities to minimise obesogenic practices, promote healthier lifestyles, and facilitate a “safe environment” for weight loss. 

## Figures and Tables

**Table 1:** Healthcare service providers’ jobs, years of experience with individuals with obesity, and location of practice.

Healthcare Service Provider	Occupation	Gender	Location of Practice	Years of Experience with Individuals with Obesity
1	Dietician	Female	Private clinic/business	8
2	Dietician	Male	Private clinic/business	2.5
3	Dietician	Female	Hospital	5
4	Nurse	Female	Hospital	13
5	Dietician	Female	Private clinic/business	9
6	Dietician	Female	Hospital	9
7	Dietician	Female	Hospital	25
8	Nurse	Female	Hospital	20
9	Dietician	Female	Hospital	10
10	Dietician	Female	Private clinic/business	2.5
11	Doctor	Male	Hospital	20
12	Dietician	Female	Private clinic/business	3
13	Nurse	Female	Hospital	25
14	Doctor	Male	Private clinic/business	23

## Data Availability

The data presented in this study are not readily available due to ethical restrictions. Requests to access the datasets should be directed to the corresponding author.

## Supplementary Materials

The following supporting information can be downloaded at: https://www.mdpi.com/article/10.3390/nu16101540/s1, Table S1: Original Chinese text of the quotes and English translation.
