# Supplementary material for: Healthcare Service Providers’ Perspectives on Sociocultural Aspects Affecting Weight Management Activities Amongst People with Obesity in Taiwan—A Qualitative Study
Source: Nutrients. 2024 May 20;16(10):1540. doi: 10.3390/nu16101540 (PMC11124406; doi:10.3390/nu16101540)
Supplement: Supplementary file 1 [file nutrients-16-01540-s001.zip › nutrients-2977279-supplementary.pdf]

Supplementary Table S1. Original Chinese text of the quotes and English translation.

Quote Number	Chinese	English
Q1	可能就會覺得說『我要減肥，就是一定是辛苦的，一定是不能吃東西的』，會把他自己設一個很難的門檻。好像就是『我就是永遠都不能再吃蛋糕了』之類的。	*"…they may think that if [they] want to lose fat, it must be hard work, certainly can't eat things. They will set themselves up with a difficult threshold. Like, "I just can't ever eat cake again", and so on."* HSP12 (dietician, private industry, 3 years of experience)
Q2	...很多時候是在解決情緒的問題...可是一有壓力就是白飯可以兩三碗，蛋糕可以吃一條...你就知道他絕對是嗯心理層面有關[的]問題，絕對不是嗯生理需求...但是這過程非常很久，因為你要他會不斷的循環，然後你要不斷的跟他講，說你心態上面要怎麼調整你要怎麼想，他可能有時候會想要放棄，我們要努力去鼓勵他。	*…a lot of time is spent on solving emotional problems… whenever they face pressure, they can eat 2-3 bowls of white rice and one entire rectangular bar of cake …you know they are definitely, um, psychologically related problems, definitely not, um, physiological needs… But this process is very long because [they] keep on continuing the cycle, then you have to continuously talk to them, how to adjust their attitude, how to think, they may sometimes think of giving up, we have to try hard to encourage them. (Q2)*<br>– HSP5, dietician, female, private clinic/business
Q3	...比如說:如果他們有曾經吃過，我會問他說，比如說，你們，你覺得這樣子的便當它的它的概念在賣什麼?他貴一定不是賣食材! 不是貴在食材，是貴在概念。...好，今天我沒有很多錢，那我沒有辦法買這種便當，那我到 7-11 去我怎麼做到 lowGI？還是可以阿!	*…. For example, if they have eaten [a healthy lunchbox] before. I will ask them, for example, you, what do you think this kind of lunchbox concept is selling? It's expensive but it isn't just the price of the ingredients. It's not expensive because of the ingredients but the concept... Well, today I do not have a lot of money, and I cannot buy this kind of lunch, then I went to 7-11, how do I achieve lowGI? It's still possible! (Q3)*<br>- HSP9, dietician, based in hospital, 10 years of experience, would guide PwO on how they can access and assemble affordable meal items mimicking healthy concepts present in the healthy lunchboxes.

Q4	…他們家的人的習慣就很相似，很多人都不是他一個人胖，是全家都還滿胖呵呵	*"…their family's habits are similar, for many people it's not only themselves that's fat, it's the whole family that's pretty fat* [chuckles]<br>HSP8 (nurse, hospital, 20 years of experience)
Q5	…她[媽媽]可能回去，她就把她們家的食物煮的很健康，然後然後也有先生就說『你吃你煮的，這食物能吃嗎？有夠難吃像狗屎一樣! 』像這樣子就會因為家人的反對的話，她可能在動機上，可能就會受到影響…	… maybe when she [mothers] goes back home, they will cook all the food really healthily, and then, and then, also have some husbands say "you eat what you cook, is this food edible? It's so unpalatable, like dog poop!" Like this, then because of the family's opposition, maybe in terms of her motivation, maybe will be influenced... – HSP8, nurse, hospital, 20 years of experience
Q6	[像]警察[一樣]的家人，就是你在吃[的時候] 『你又在吃，你不是在減重嗎？你怎麼可以吃這個東西？』這叫警察，那或許如果家人是比較 nice 的，跟他、請他說『喔，你現在吃這個...那我們來看看欸』...或許說「你真的餓了嗎？」、『需不需要我幫忙你看看要不要怎麼控制或是什麼』，那個是不一樣的話語，溝通的話語是不一樣，所以對病人的心態上面也是不一樣的。	Police-like family members [who closely monitor you], when you are eating, "You're eating again, aren't you losing weight? How can you eat this thing?", this is called policing. Maybe if your family is nicer, tell them or ask them to say, "Oh, you eat this now... Then let's take a look"... or maybe say "Are you really hungry?", "Do you need me to help you see if you want to [know] how to control or something", that is different, the words and communication words are different, so it's different on the mentality of the patient. (Q6)<br>- HSP7, dietician, based in a hospital, 25 years of experience
Q7	對，其實從十二月開始都是了，十二月到過年後一個月都會，因為畢竟這時候是聖誕節嘛、跨年嘛、尾牙、過年、春酒，哇! 全部節慶	"*from December to one month after the Lunar New Year* [usually occuring between mid January to mid February]*, because after all, at this time it's Christmas, New Year's Eve, year end banquet [traditional annual company event to show appreciation for staff], New Year, spring feast [company event around Lunar New Year], wow! All celebrations*" (Q7)<br>HSP10 (dietician, private clinic/business, 2.5 years of experience)
Q8	你看滿街都在跑，我記得滿街都在跑，應該是這一、兩年的事情	*"you see all the streets are filled with [food delivery workers]*

		HSP7 (dietician, hospital 25 years of experience)
Q9	…以前我們常常說我的病人要減重，他要有一個安全的環境，但臺灣的美食很多，其實這對病人的挑戰性是很高的，那尤其臺灣的食物，小吃很多，那，那這個...對我們的病人就是年紀大一點的病人，他有印象中他的美食的文化、他的傳統飲食，那傳統飲食裡面可能就高油、高糖，可是那是他的過去的飲食的經驗，是他喜歡的，那個也會造成病人就是很難去達到想要減輕體重的時候，但是這些食物對他來說又有他的意義在。	…We used to often say that when my patient wants to lose weight, they need a safe environment, but there are a lot of delicacies in Taiwan. Actally, this is very challenging for patients. Especially as Taiwanese cuisine has a lot of street foods Note: street foods tend to be classified as junk food due to preparation methods and are commonly referred to as a subsection of Taiwanese delicacies]. Then, then this... to our patients, like older patients, in their impression of the food culture, their traditional diet, the traditional diet may be high in oil and sugar, but that is their past dietary experience, which they like, that will also make it difficult for patients to achieve their desired weight loss. But at the same time, but these foods also have meaning to them. (Q9)<br>- HSP7, dietician, hospital 25 years of experience
Q10	那再來就是西方人的那個飲食他們基本上是一個 plate，他們就是我這個一份、這個一份，他們是用份量在算的，跟台灣的是不一樣的，我們台灣其實沒有那個 plate 習慣，我們就是一桌菜，就是大家我拿我的碗這樣夾來吃… 我吃完再夾、吃完再夾，所以我不知道後來我吃了多少，是這種生活型態…在美國他 my plate 他可以推，在台灣是不行，但是現在我們開始在推了，就是說你要吃的東西都是先一起拿之後就停了，就不能再拿了…這個部分也是很多是不一樣… 不過原則性的東西是一樣的，比如說要 low carbohydrate 然後 high protein 那些等等…	Then also, the Westerners their diet is basically a plate, they're like, this is one portion, that is one portion, they are using portions to calculate, this is different in Taiwan. In Taiwan we don't have this plate habit, we just have a table of dishes, and everyone takes their own bowl and take what they want to eat … after I finish [the small serves of each dish], I grab more, finish eating, then grab more, so later I do not know how much I ate, it's this kind of [eating] pattern… in the United States MyPlate can be promoted but can't in Taiwan, but now we are starting to promote it, so what you want to eat, just take all [the dishes you want to eat] together [in one go] and then just stop, you cannot take anymore…a lot [of practices are] not the same… but the things in principle are the same, for example, to have low carbohydrate and high protein those, and so on… (Q10)<br>- HSP11, doctor, hospital, 20 years of experience

Q11	常聽那個BMI大於35以上的，常常會問我們一件事情， 他是說，『有沒有那一種不用太麻煩就可以減重的事情，不用做任何改變我就可以減重這種事情? 』，所以為什麼現在網路上面會那麼多減重模式就是他會說:你不用，不用那麼辛苦，你就可以減重幾公斤。...對於想減重的人是一個需求跟渴望，對，但是他們其實都一直沒有辦法接受，需要因為努力才能夠減重的這個過程...	…I often hear a person with a BMI greater than 35, often ask us one thing, they ask "Is there such a thing that you can lose weight without too much trouble, something with no need to change anything and then I can just lose weight?", so that's probably why there are so many weight loss methods on the internet like they would say that you don't, don't have to work so hard, and you can lose how many kilos…For people who want to lose weight, it is a need and desire, yes, but continuously, they actually have not been able to accept that, there is a need for hard work then they would be able to go through the process of losing weight… (Q11)<br>-HSP1, dietician, private clinic/business, 8 years of experience
Q12	所以當然要先跟他釐清楚他聽到的是什麼，有些東西其實是OK的，那你就說OK，因為減重最好的方法就是他可以接受的方法...	*"so of course you must first clarify with them what they heard, some things are actually OK, then you just say OK, because the best weight loss method is the method that they can accept…"* (Q12)<br>HSP11 (doctor, based in hospital, 20 years of experience) summarizes the approach the HSPs in the study had,
Q13	嗯...體重比較重的人，光是叫他進健身房他其實就會害怕， 也有[會害怕別人的眼光]，然後『我不會用這些東西，看起來很難，這個感覺我都不適合』，我覺得那個心理的害怕...那個就會害怕了，更何況他們對身體或多或少還是會有一點覺得，『啊，我的體重上面是比較重的』	*Hmm... a person with heavier weight, just telling them to go into the gym, they, in fact, would be afraid, also [will be afraid of people seeing them], then "I don't know how to use these things, looks very difficult. I don't feel like this suits me." I think that psychological fear… that would be scary, not to mention that towards their body, they still more or less will feel a little, "ah my weight is heavier [than others] (Q13)*<br>- HSP6, dietician, hospital, 9 years of experience
Q14	...因為長期他才會養成『我現在的作息是這樣，所以我家人的飲食就是這樣，我朋友的飲食也是這樣，所以我長期我就不會運	*… because long-term they would develop like, "my habit is now like this, so my family diet is like this, my friend's diet is also like this, so long term-wise I don't*

	動，那所以你要突然我身邊多出一個會運動的人』，我覺得這個可能也比較不是那麼容易啊…我之前也遇過[一個患者]。她就說嗯…我叫她運動，她說:『嗯…可是我要跟我男朋友約會，我男朋友不要運動。我們兩個要去吃好吃的, 那如果我去運動，我們兩個就少了約會的時間』	*exercise, then you want me to suddenly have people who exercise in my life", I think this may be relatively not so easy ah…I have encountered [a patient]... I told her to exercise. She said "Um… but I want to date my boyfriend, my boyfriend doesn't want to exercise. The two of us want to go eat delicious food, then if I go to exercise, the two of us will have less time to date" (Q14)*<br>- HSP6, dietician, hospital, 9 years of experience
Q15	因為工作時間可能八點到六點，甚至有些行業會弄到七點多，更晚。那所以對於他們來說，回家吃個飯，其實就差不多要休息了。	*"because working hours may be from 8 to 6, and even in some industries, it will get past 7 o'clock, even later. So, for them, [after] going home to have a meal, in fact, it's about time to have to rest."* (Q15)<br>HSP6 (dietician, based in hospital, 9 years of experience) recalls her observations
Q16	她說，因為很多人都叫他去運動。很多醫護人員都叫她一定要去，去健身房運動，可是她跟我說『不是我不要』… 她覺得很難過。…因為她說，他的器具不是for百公斤以上的人，會受傷。…我們就先在家裡做一些一些動作，那你去找復健科醫師，請他教你一些撇步，然後你就可以自己做。所以他後來去download一些YouTube上面的一些很簡單的一些體操他就在家裡做。	*[A patient] said, because many people told her to exercise. Many medical staff told her she must go, go to the gym to exercise, but she told me "it's not that I don't want to"…she felt very sad…because she said, [the gym] equipment is not for people more than one hundred kilograms, they'll get injured… [I suggest she] do some exercises at home first. Then you go to the rehabilitation physician and ask them to teach you some steps. Then you can do it yourself. So she later went to download some very simple body exercises from YouTube and they did it at home (Q16)*<br>-HSP9, dietician, based in hospital, 10 years of experience
Q17	…光是小學就會犧牲體育課去念書了啊!	*"…even elementary schools will sacrifice physical education to go study!"* (Q17)<br>(HSP12, dietician, private clinic/business, 3 years of experience)
Q18	我不知道是不是跟跟以前台灣早期,像我爸媽那個年代...對於他們來說沒有什麼去運動這種休閒。他們覺得運動好像是一個休	*I don't know if it is the same as the early days of Taiwan, like my parents' era … For them, there isn't say any leisure to exercise. They feel like sports is*

	閒娛樂，不是生活裡面應該要有的東西。生活裡面就是，上班、回來、休息、準備明天、睡覺、明天上班。	*recreational entertainment, which isn't something that is necessary in life. In life, it's just, go to work, come back, rest, and prepare for tomorrow, go to bed, go to work tomorrow (Q18)*<br>-HSP6, dietician, based in hospital, 9 years of experience
Q19	…大部分的人都是在十二點以後才睡覺，甚至一點ㄟ。… 然後一定會影響到你的賀爾蒙, 然後另外一方面，現在人生活壓力大、工作壓力大、然後又或者是長期的生活習慣不好，導致睡眠品質越來越不好。而且就我們診所的經驗來說，最難調的就是睡眠… 不知道是生活壓力還是生活習慣不好，就是就是就是摸啊，或是我就覺得十二點睡很早欸呵呵…	*… most people sleep after midnight, or even 1 [am] eh! … then it will definitely affect your hormones, and on the other hand, people now have a lot of pressure in life, lots of work pressure, and also-, or bad long-term living habits, which leads to sleep quality getting worse and worse. And with our clinic's experience, the most difficult to adjust is sleep … I do not know if it's life stress or bad lifestyle habits, that is, that is, fiddling around or [thinking] "I think twelve o'clock is too early to sleep eh" … (Q19)*<br>-HSP5, dietician, private clinic/business, 9 years of experience
Q20	大部分是他們自己可以嘗試去調整	"*most of them can try to adjust by themselves*" (Q23)
Q21	是工作？家裡？還是是你個人的情緒？還是就是什麼分手那些等等的，那他們自己就會說了。那如果要再更深入一點的，說實在的，我真的也幫得不多？!	*Is it work? Home? Or is it your personal emotions? Or if it's just due to breaking up and so on, then they will say it themselves. Then if you want to go a little deeper, to be honest, I really can't help much?* ! *(Q20)*<br>-HSP10, dietician, private clinic/business, 2.5 years of experience
